# Catecholamine and Volume Therapy for Cardiac Surgery in Germany – Results from a Postal Survey

**DOI:** 10.1371/journal.pone.0103996

**Published:** 2014-08-01

**Authors:** Christoph Sponholz, Christoph Schelenz, Konrad Reinhart, Uwe Schirmer, Sebastian N. Stehr

**Affiliations:** 1 Department of Anesthesiology and Critical Care Medicine, University Hospital Jena, Jena, Germany; 2 Integrated Research and Treatment Center, Center for Sepsis Control and Care (CSCC), Jena University Hospital, Jena, Germany; 3 Institute of Anaesthesiology, Heart and Diabetes Center NRW, Ruhr University of Bochum, Bad Oeynhausen, Germany; San Raffaele Scientific Institute, Italy

## Abstract

**Background:**

Management of cardiac surgery patients is a very standardized procedure in respective local institutions. Yet only very limited evidence exists concerning optimal indication, safety and efficacy of hemodynamic monitoring catecholamine and fluid therapy.

**Methods:**

Between April and May 2013, all 81 German anaesthesia departments involved in cardiac surgery care were asked to participate in a questionnaire addressing the institutional specific current practice in hemodynamic monitoring, catecholamine and volume therapy.

**Results:**

51 (63%) questionnaires were completed and returned. All participating centers used basic hemodynamic monitoring (i.e. invasive arterial blood pressure and central venous pressure), supplemented by transesophageal echocardiography. Pulmonary arterial catheter and calibrated trend monitoring devices were also routinely available. In contrast, non-calibrated trend monitoring and esophageal doppler ultrasound devices were not commonly in use. Cerebral oximetry is increasingly emerging, but lacks clear indications. The majority of patients undergoing cardiac surgery, especially in university hospitals, required catecholamines during perioperative care, In case of low cardiac output syndrome, dobutamine (32%), epinephrine (30%) or phosphodiesterase inhibitors (8%) were first choice. In case of hypotension following vasoplegia, norepinephrine (96%) represented the most common catecholamine. 88% of the participating centers reported regular use of colloid fluids, with hydroxyethyl starches (HES) being first choice (64%).

**Conclusions:**

Choice of hemodynamic monitoring is homogenous throughout German centers treating cardiac surgery patients. Norepinephrine is the first line catecholamine in cases of decrease in peripheral vascular resistance. However, catecholamine choice for low cardiac output syndrome varies considerably. HES was the primary colloid used for fluid resuscitation. After conduct of this survey, HES use was restricted by European regulatory authorities in critically ill patients and should only be considered as second-line fluid in surgical patients without renal impairment or severe coagulopathy. Large clinical studies addressing catecholamine and fluid therapy in cardiac surgery patients are lacking.

## Introduction

An estimated 100.000 cardiac surgical procedures are performed each year in Germany [1]. Intraoperative anesthesiological management of cardiac surgery patients has developed to a point where complex monitoring tools and differential catecholamine and volume therapy are routinely used.

Despite the fact that the intraoperative anesthesiological approach to cardiac surgical procedures are usually standardized within the setting of the respective institutions, very little to no clinical data is available concerning appropriate intraoperative hemodynamic monitoring, vital parameter goals, and catecholamine and volume therapy in cardiac surgery patients [2]–[6]. Only few small clinical trials for instance compare the effects of catecholamines during and immediately after cardiac bypass surgery [7]–[9]. In December 2013, triggered by large-scale clinical trials which demonstrated the lack of benefit and increased harm after use of HES in critically ill and septic patients [10], [11], European regulatory authorities restricted HES use in critically ill patients and issued major warnings for use in surgical and trauma patients. In these settings, HES should only be used if crystalloids are not sufficient to treat hypovolemia. HES use is contraindicated in patients with increased risk of renal failure and bleeding.

In 2005 the German Society for Thoracic and Cardiovascular Surgery (DGTH) and the German Society for Anaesthesiology and Intensive Care Medicine (DGAI) initiated and then updated S3 guidelines for postoperative intensive care in cardiac surgery patients [12], [13].

We present the results of a postal survey evaluating the current intraoperative practice regarding hemodynamic monitoring, catecholamine and volume therapy at German cardiothoracic anaesthesia centers. The results of this survey could serve as a basis for the development of guidelines for the intraoperative care of cardiac surgery patients.

## Methods

### Ethics

The study was approved by Jena University Ethics Committee which waived informed consent because of the anonymous nature of the study.

### Data collection

A postal questionnaire was sent by the DGAI to the department heads of the 81 institutions that have a cardiac surgery department in Germany. There was a covering letter explaining the aims of the study and a stamped addressed return envelope for return postage. The letters were sent to the hospitals in the period between 01 April 2013 and 31 May 2013. All letters were delivered by mail, and no letters were returned because of an invalid address. Due to the fact that the acquisition of the data was performed anonymously and the questionnaires were collected by the society, no estimate of survey characteristics for nonrespondents and respondents can be made to assess the potential nonresponse bias.

### Questionnaire

The questionnaire consisted of 23 questions covering four major areas: a) structural data regarding hospital structure and patient care, b) standard procedures of hemodynamic monitoring as well as implementation of advanced regional perfusion monitoring devices, c) use of first- and second line vasoactive agents or inotropic drugs in hypotension following low cardiac output syndrome or vasoplegia and d) different volume replacement strategies, with a special focus on colloidal fluids and crystalloids. Frequencies in the use of different monitoring devices or volume replacement were categorized on a one to five graded Likert scales ranging from one - always present/is always true to - five - very rare/not present. The questionnaire itself is provided in File S1. The questionnaire was filled out and returned anonymously to the society.

Evaluation of the questionnaires were performed anonymously after having collected all the returned sheets. Due to the study design all missing values represented missing answers. Categorical data were assessed and depicted by frequencies. Values graded on Likert scales were analyzed by descriptives (median, mean, minimum and maximum values as well as 95% confidence intervals) calculated with IBM SPSS statistics Version 21.

## Results

### Structural and hospital data

51 of the 81 (63%) institutions with a cardiac surgery department in Germany answered and returned the questionnaire. Of these, 50 questionnaires were eligible for further evaluation. One questionnaire from a pediatric cardiac surgery department was excluded from further analysis, because of specific pediatric operative procedures and pathophysiology that could possibly influence fluid administration and catecholamine use.

**Figure 1:**
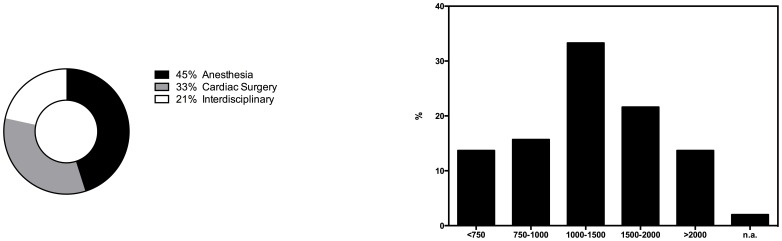
Overview of the departments responsible for postoperative intensive care of cardiac surgery patients (in percent) A (left). Number of operations performed in the respective cardiac surgery departments per year **B (right)**.

Table 1 shows the participating cardiac surgery departments and the level of hospital care. Postoperative intensive care units (ICU) were predominantly managed by anesthesiologist (n = 22 (44%)), followed by cardiac surgeons (n = 17 (34%)) or were organized by an interdisciplinary team (n = 11 (22%), **figure 1A**).

**Table 1:** Overview of the type of surgical center that the 51 cardiac surgery departments are associated with.

	N (%)
Heart Center	16 (31)
University Hospital	18 (35)
Maximal Care Hospital	15 (29)
Children's Heart Center	1 (2)
Not specified	1 (2)

17 (34%) centers conducted 1000 to 1500 cardiac operations per year, 11 (22%) centers between 1500 and 2000 and 7 (14%) more than 2000 operative procedures per year. 8 (16%) and 6 (12%) centers performed between 750 and 1000 or less than 750 cardiac operative procedures per year, respectively (**figure 1B**). A detailed break down of surgical procedures revealed that the median proportion of coronary bypass surgery was 50% (mean: 50.81, 95% CI 46.89–54.73) and the median use of intraoperative cardiopulmonary bypass (CPB) was 90% (mean: 82.19; 95% CI 77.67–86.71).

### Measurement of macrohemodynamics and regional perfusion

**Figure 2:**
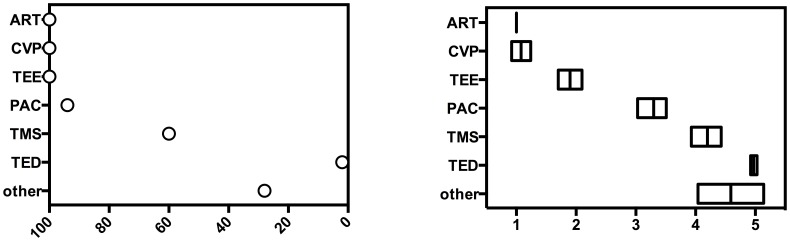
Availability of monitoring devices (in percent) A (left) and actual use of these devices B (right) (1–5 Likert scale, where 1 is common, 5 rare, ART = arterial blood pressure measurement, CVP = central venous pressure, TEE = transesophageal echocardiography, PAC = pulmonary arterial catheter, TMS = calibrated trend monitoring system, TED = uncalibrated trend evaluation systems).

All centers participating in this survey (n = 50 (100%)) used intraoperative transesophageal echocardiography (TEE) to monitor macrohemodynamic parameters in addition to bedside basic monitoring (i.e. invasive arterial pressure and central venous pressure, **figure 2A**). Pulmonary arterial (PA) catheters were commonly available (n = 47 (94%)), as were calibrated trend monitoring devices (i.e. PICCO-System, Pulsion, Germany, n = 30 (60%)). Uncalibrated trend monitoring systems (i.e. Vigileo, Edward Lifesciences, USA) or esophageal doppler sonography (i.e. CardioQ, Deltex Medical, UK) were only seldom available 28% (n = 14) and 2% (n = 1), respectively. Two centers reported the intraoperative use of transcranial doppler sonography (TCD) or left atrial pressure (LAP) measurement, respectively.

The frequency of intraoperative macrohemodynamic monitoring was assessed with a one to five categorical Likert scale. All centers (n = 50 (100%)) reported the use of basic monitoring (i.e. invasive arterial pressure measurement). With a median value of 1 (mean: 1.08, 95% CI: 0.92–1.24) measurement of central venous pressure (CVP) was also a common parameter of intraoperative macrohemodynamic monitoring. Moreover, TEE was frequently used with a median value of 2 (mean: 1.9, 95% CI 1.7–2.1), followed by PA catheterization (median value: 3, mean value: 3.3, 95% CI 3.03–3.51) and calibrated trend monitoring systems (median value: 4, mean: 4.2; 95% CI: 3.93–4.43). The actual application of uncalibrated trend monitoring devices (median value: 5,mean value: 4.56; 95% CI: 4.28–4.83), transesophageal doppler sonography (median value: 5, mean value: 4.97; 95% CI: 4.92–5.03) or other devices (i.e. TCD or LAP) (median value: 5, mean value: 4.59; 95% CI: 4.04–5.14) only played a minor role in measuring intraoperative hemodynamics (**figure 2B**). Regional organ perfusion monitoring in cardiac surgery patients was also a part of the questionnaire. 37 (74%) of the participating centers used cerebral oximetry monitoring, followed by continuous mixed venous (SvO2, n = 16 (32%) or central venous (ScvO2, n = 6 (12%)) saturation monitoring. While 6 (12%) of the centers mentioned the use of other applications for regional perfusion monitoring (i.e. intermittent SvO2 (n = 2), intermittent ScvO2 (n = 2) or TCD (n = 2)), no centers used intraoperative gastric tonometry measurement. 9 (18%) of the participating centers never used any form of intraoperative regional perfusion monitoring. Quantified on a one to five categorical Likert scale usage of intraoperative cerebral oximetry yielded a median value of 3 (mean value: 3.24; 95% CI: 2.90–3.58). All other devices for regional perfusion measurement had a median score of five on the Likert scale and were therefore very rarely used.

### Perioperative use of catecholamines

**Figure 3:**
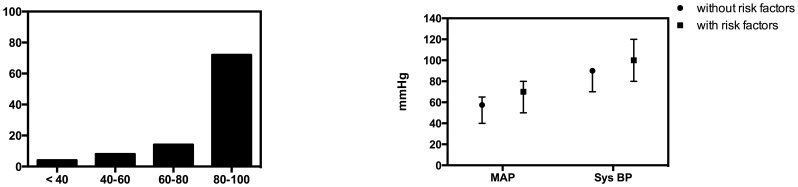
Percentage of patients requiring catecholaminergic therapy in the perioperative period A (left) and target mean arterial pressure (MAP) and systolic blood pressure (Sys BP) in cardiac surgery patients with or without risk factors B (right).

36 (72%) of all returned questionnaires reported that 80–100% of patients received catecholamines within the perioperative period. Another 7 (14%) centers reported 60–80% and 4 (8%) of 40–60% catecholamine dependency in patients during perioperative care. Only 2 (4%) centers reported that less than 35% of patients needed catecholamines (**figure 3A**).

Standard operating procedures (SOP) for intraoperative catecholamine use were available in 23 (46%) of the participating centers, while 25 (50%) had no SOP for intraoperative catecholamine use. In the postoperative period 18 (36%) centers applied an existing SOP for catecholamine administration, while 22 (44%) centers had no such SOP. In 20% the availability of a SOP for postoperative catecholamine use remained unknown (n = 6, in cases of postoperative surgical ICU care). Nevertheless, 62% of the participating centers applied fluid challenges and/or used catecholamines in case of decreasing mean arterial blood pressure (MAP) or systolic blood pressure (systBP).

Seven centers reported the use of intraoperative fluid challenges or catecholamines in case of decreasing MAP below 60 mmHg (mean: 61.43; 95% CI 59.17–63.68) or systBP below 80 mmHg (n = 2). In cases of absent severe comorbidity (i.e. stenosis of the carotid artery or long lasting arterial hypertension) lower median MAP limit (n = 18) or systBP (n = 6) was 57.5 mmHg (mean: 55.28; 95% CI 51.92–58.63) or 90 mmHg (mean: 85; 95% CI 76.22–93.78), respectively. On the other hand, when severe comorbidity was present, the lower median MAP limit (n = 18) or systBP (n = 5) increased to 70 mmHg (mean: 67.22; 95% CI 63.68–70.76) or 100 mmHg (mean: 102; 95% CI 83.58–120.42), respectively (**figure 3B**).

**Figure 4:**
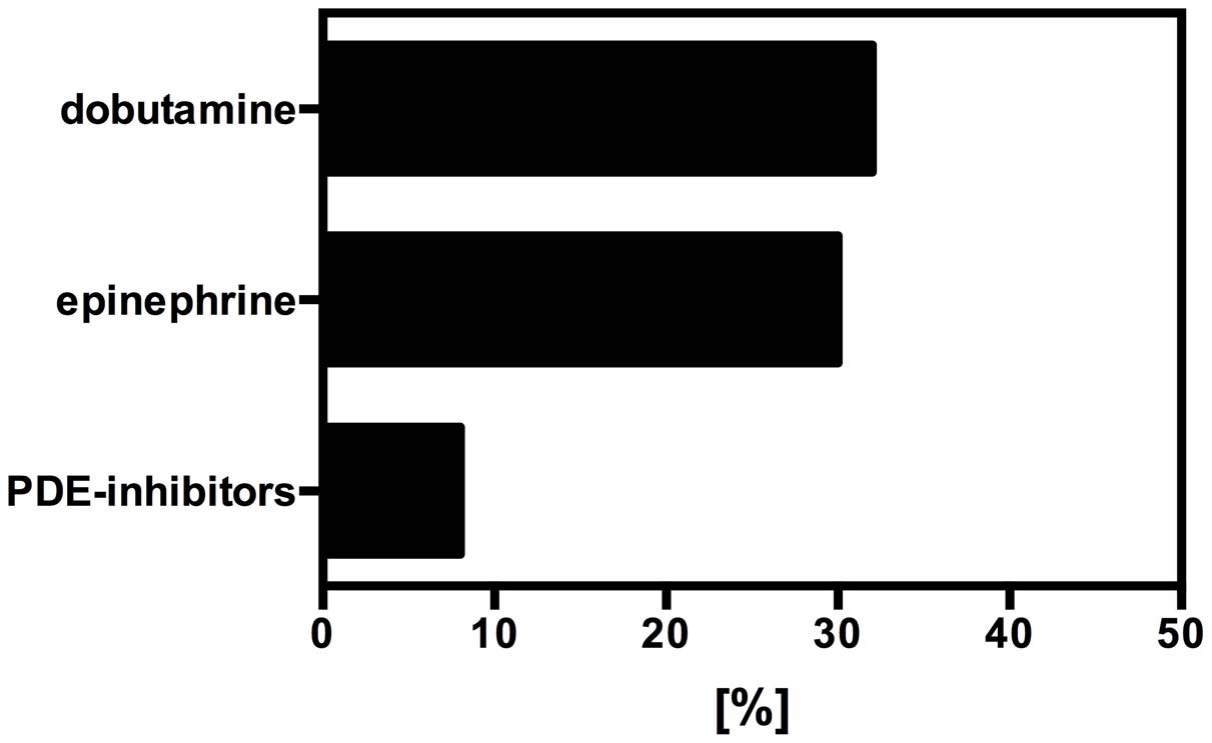
First line catecholamine therapy for low cardiac output (in percent).

In case of hypotension caused by low cardiac output the most commonly used catecholamine was dobutamine (n = 16 (32%)), followed by epinephrine (n = 15 (30%)) and phosphodiesterase inhibitors (PDE-inhibitors (n = 4 (8%)). All other substances were rarely used (**figure 4**).

**Figure 5:**
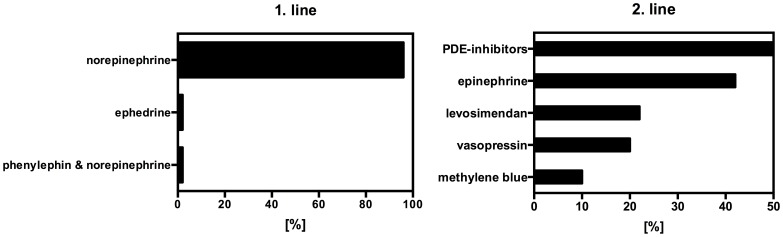
First line catecholamine therapy for vasoplegia A (left) and second line catecholamine choice in percent B (right).

48 (96%) of all centers used norepinephrine as the first line catecholamine in patients with hypotension caused by vasoplegia, followed by ephedrine (n = 1 (2%) or phenylephrine+norepinephrine (**figure 5A**, n = 1 (2%)). In cases where the first line catecholamines fail in case of hypotension caused by low cardiac output or vasoplegia, most centers preferred PDE-inhibitors (n = 25 (50%)) or epinephrine (n = 21 (42%)) as second line catecholamines, followed by levosimendan (n = 11 (22%)), vasopressin (n = 10 (20%)), norepinephrine and methylene blue (**figure 5B**, n = 5 (10%), each). Interestingly, 6 (12%) centers reported to be influenced in their particular choice of catecholamines, e.g. by surgeons.

### Perioperative fluid management

**Figure 6:**
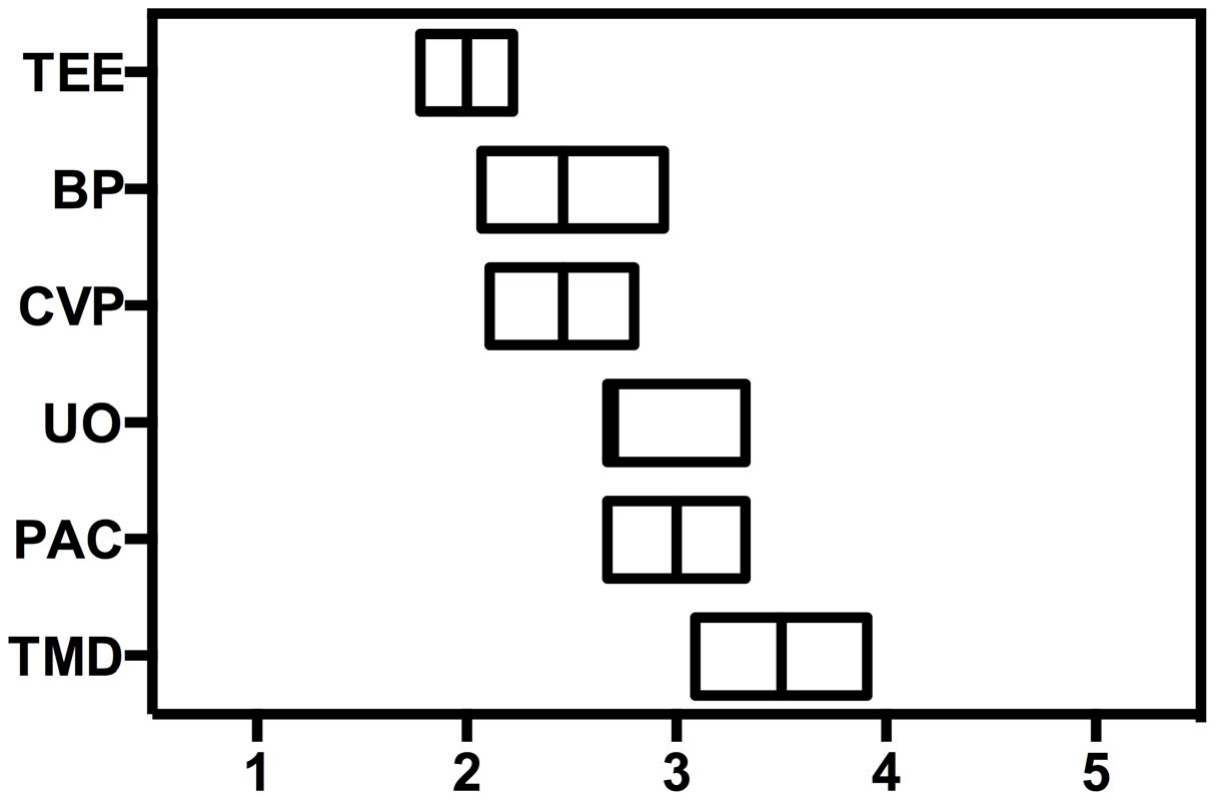
Monitoring for perioperative volume therapy (1–5 Likert scale, where 1 is common, 5 rare, TEE = transesophageal echocardiography, BP = blood pressure, CVP = central venous pressure, UO = urine output, PAC = pulmonary arterial catheter, TMD = trend monitoring devices).

Useful parameters to indicate volume deficiency remain controversial. Indicated on a one to five categorical Likert scale clinical sings of volume deficiency (i.e. undulating blood pressure curve) (median value: 2, mean: 2.46; 95% CI: 2.07–2.84), right ventricular filling pressures (i.e. CVP) (median value: 2, mean: 2.46; 95% CI: 2.11–2.80) or TEE (median value: 2; mean: 2.0; 95% CI: 1.78–2.22) were predominantly used for monitoring of fluid administration. Urine output (median value: 3, mean: 2.7; 95% CI: 2.36–3.05), PA occlusion pressure (median value: 3, mean: 3.0; 95% CI: 2.67–3.33) and trend monitoring systems (i.e. PICCO or Vigileo) (median value: 3.5, mean: 3.5; 95% CI: 3.09–3.91) were more seldom used (**figure 6**).

**Figure 7:**
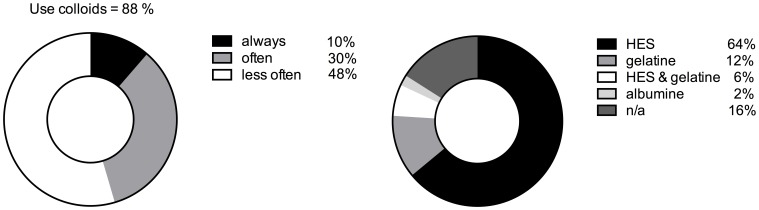
intraoperative use of colloids A (left) and type of colloid solution used in percent B (right). n/a – not applicable in case colloidal fluids were generally avoided in the respective institution.

88% of the participating centers reported regular colloid use in their patients (**figure 7A**). Hereof, 5 (10%) centers stated to nearly always use colloids, 15 (30%) to make often and 24 (48%) less often use of colloids. In 6 (12%) reported no use of colloids what so ever. Within the group of colloids hydroxyl ethyl starches (HES) substances were of first choice in 64% (n = 32) of the participating centers, followed by gelatine (n = 6 (12%), HES and gelatine in combination (n = 3 (6%) and albumin (n = 1 (2%). 7 (14%) centers try to avoid intraoperative colloidal fluid administration (**figure 7B**). Crystalloid fluid administration scored 2 in median (mean: 2.62; 95% CI 2.11–3.13), while HES substances and fresh frozen plasma (FFP) were placed in median on 3 (mean 3.45; 95% CI: 3.09–3.81) and 4 (mean: 3.81; 95% CI: 3.53–4.10), respectively. Gelatine and albumin were of minor choice, reaching median values of 5. The same pattern could be found in postoperative fluid management, with crystalloid infusion reaching a median score of 2 (mean: 2.88; 95% CI: 2.29–3.47), followed by HES containing substances with a median score of 3 (mean: 3.13; 95% CI: 2.71–3.54) and FFP with a median score of 4 (mean: 3.54; 95% CI: 3.20–3.88). Again, gelatine and albumin were of minor choice with a median score of 5.

**Figure 8:**
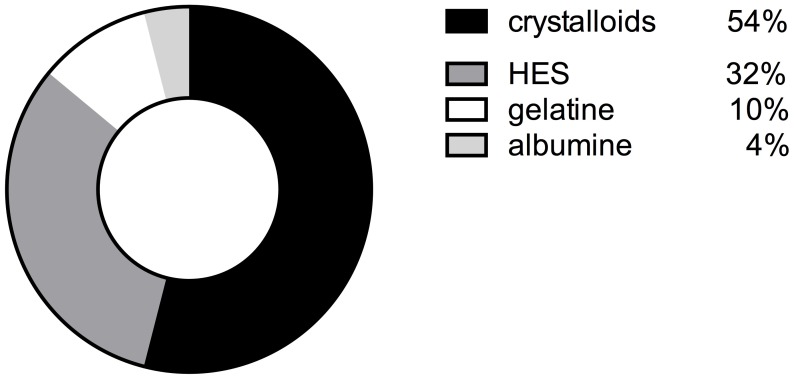
Volume used for cardiopulmonary bypass priming in percent.

Cardiopulmonary bypass priming was mainly performed with pure crystalloid fluids in 54% (n = 27), while 32% (n = 16) of the participating centers used HES fluids. In 10% (n = 10) gelatine or albumin (4% (n = 2)) was used for priming (**figure 8**).

58% (n = 29) and 44% (n = 22) of all participating centers applied a SOP for intraoperative or postoperative erythrocyte transfusion, respectively.

Catecholamine- and volume therapy substantially varied between the different levels of hospital care. Calibrated trend monitoring devices seemed to be more commonly available in university and maximal care hospitals in comparison to specialized heart centers (**table S1 in File S2**). However, intraoperative use of calibrated trend monitoring devices were almost equally ranked on the 1 to 5 Likert scale among the different level of the participating centers (**table S2 in File S2**). Intraoperative regional perfusion monitoring with cerebral oximetry devices were equally distributed among all centers, whereas continuous mixed venous or central venous saturation monitoring were almost exclusively available in university hospitals (**table S3 in File S2**). Specialized heart centers or maximal care hospitals more frequently applied no intraoperative regional perfusion monitoring device whatsoever (**table S4 in File S2**).

The participating university hospitals reported that 80–100% of all patients had some form of catecholamine therapy (supplemental **figure S1 in File S2**). Interestingly, 4 (22.2)% of all participating cardiac anesthesia departments located at university hospitals reported to be influenced in their particular choice of catecholamines, e.g. by the surgeons (**table S5 in File S2**).

Among all participating centers, postoperative fluid administration was mainly conducted with crystalloids, while intraoperative colloidal fluid administration was much less common among university hospitals (**table S6 in File S2**), Specialized heart centers and maximal care hospitals mainly performed cardiopulmonary bypass priming exclusively with crystalloids, while almost 60% of the participating university hospitals used colloidal fluids as priming solutions (**table S7 in File S2**). Furthermore, specialized heart centers more frequently applied standard operating procedures for intraoperative (81%) or postoperative (75%) red blood cell transfusion, while this was less common among the participating university and maximal care hospitals (**table S8 in File S2**).

## Discussion

In this study, we document the wide diversity of approaches towards monitoring, catecholamine application and volume therapy among German departments of cardiac anesthesia. The most striking result of our questionnaire is the divergent choice of catecholamines for the treatment of a perioperative low cardiac output syndrome. Cardiothoracic surgery is a highly standardized professional discipline in Germany, with performance of around 100.000 operative procedures every year [1]. It is therefore surprising that important intra- and postoperative approaches, especially regarding fluid and catecholamine administration, differ considerably from department to department, as shown in this survey.

In general, monitoring tools of macro- and regional hemodynamic parameters are generally used and play a central role in perioperative care in this special patient cohort, not only since the revision of the S3 guideline for intensive care in cardiac surgery patients by Carl and colleagues in 2010 was published [13]. It is therefore not surprising that all centers use basic monitoring, supplemented by CVP measurement. Moreover, every single center routinely uses TEE. PA catheterization and calibrated trend monitoring complete the scope of tools for macrohemodynamic monitoring, but are infrequently used.

Comparing our data with previous surveys in cardiothoracic surgical patients [14], [15] shows that the use of TEE seems to have increased at the expense of PA catheterization procedures.

Beside macrohemodynamic measurements, regional perfusion monitoring systems have become more and more common in perioperative care of critical ill patients [16]. The participating centers – especially university hospitals - favour continuous measurement of central- or mixed-venous saturation and the use of cerebral oximetry measurement systems. Nevertheless, widespread routine use of these systems was not evident. Instead, extended monitoring was available, but restricted to specific indications (i.e. carotid or aortic surgery).

Reviewing the literature, continuous central- or mixed-venous saturation measurement [17] as well as cerebral oximetry [18] may both be valuable tools in monitoring regional perfusion, but need further clinical evaluation to gauge their impact on patient outcome [19].

The majority of patients undergoing cardiac surgery require inotropic or vasopressor support during the perioperative phase. This may be caused by either low cardiac output, volume deficiency or lack of systemic vascular resistance. Catecholamines use was reported to be very common among university hospitals. The reasons for this finding remain speculative, but it can be assumed that perhaps a higher proportion of patients with complex cardiac diseases and comorbidities present at university hospitals rather than heart centers or maximal care hospitals. Up to date, monitoring of systolic or mean arterial pressure is currently the most commonly used clinical parameter for fluid or catecholamine administration. More than 60% of the centers answering the questionnaire initiate an intervention, either by fluid and/or catecholamine administration, by evaluating systolic blood pressure variation. Based on the presence of comorbidities, tolerable mean arterial blood pressure or systolic blood pressure values ranged between 55–67 mmHg or 85–102 mmHg, before initiation of an intervention.

Dobutamine and epinephrine were the first choice, followed by PDE-III inhibitors in case of hypotension caused by low cardiac output syndrome. In case of failing responsiveness to this first line therapy, PDE-III Inhibitors were the second line choice, followed by epinephrine and levosimendan or vasopressin. This is in accordance with previous surveys regarding the use of catecholamines in cardiothoracic surgery patients. In 2006 Kastrup and colleagues published a postal survey asking for the first line catecholamine used in postoperative low cardiac output syndrome (LCOS) in Germany. Epinephrine (41.8%), dobutamine (30.9%) and PDE-III inhibitors (14.5%) were the most common drugs [15]. In a follow up survey in 2008 epinephrine still was the first line catecholamine used for the treatment of LCOS in cardiothoracic surgery patients [14]. Williams and colleagues investigated the use of catecholamines in patients with LCOS after coronary artery bypass grafting in a high risk patient population and found a high inter-hospital variability in the use of vasoactive agents with similar patient outcome [20]. The S3 guideline for intensive care in cardiac surgery patients [13] as well as European recommendations for management of heart failure in cardiac surgery patients [21] both recommend use of catecholamines in the management of LCOS, including epinephrine, dobutamine and PDE-III inhibitors (among others), without advocating one specific substance. However, several clinical trials with patients cardiogenic [22] and septic shock [23], [24] have shown that epinephrine is inferior concerning lactic acidosis, tachycardia/arrhythmia and gastric mucosal perfusion. Therefore, the administration of dobutamine plus norepinephrine appears to be preferable, but more clinical studies are warranted in this respect. In case of vasoplegia almost all responding centers in the present survey (96%) consistently and quite frequently use norepinephrine infusion as the first line catecholamine to restore adequate perfusion pressures. This is again in line with previous surveys regarding this issue in cardiac surgical patients by Kastrup and colleagues [14], [15]. The role of norepinehprine infusion in case of vasoplegia has been validated in various studies, including severe infection/sepsis, systemic inflammatory response syndrome (SIRS) and postperfusion syndrome after cardiac surgery [25]–[28] and thus is also recommended as the first and only vasopressor in the S3 guideline for intensive care in cardiac surgery patients [13].

The wide range of catecholamine approaches in LCOS is certainly an indicator of the lack of sound clinical data pointing towards an ideal drug therapy. Hence, more clinical studies are needed to investigate catecholamine use in LCOS in patients undergoing cardiac surgery.

When this survey was conducted, the latest S3 guideline for intensive care in cardiac surgery still recommended both crystalloids and HES or other colloids for postoperative care patients [13]. Accordingly, crystalloids were the fluids of first choice during and after surgery, but HES solutions, followed by gelatin and albumin, were also commonly used during the perioperative phase in most of the centers. On the other hand, it is noteworthy that in most centers CPB priming was maintained without colloids, even if colloids were otherwise applied.

Volume replacement strategies in cardiac surgery have changed during the last years, reflecting increasing doubt about the efficacy and safety of HES solutions [14]. Large-scale clinical trials found that also “modern” third generation HES solutions increased need for renal replacement therapy (RRT) and transfusion of allogeneic blood products in critically ill patients and patients with sepsis, and increased 90-day mortality in patients with sepsis [10], [11]. A large observational study with over 6000 patients found increased need for RRT among patients undergoing cardiac surgery who received either HES or gelatin compared to crystalloids [29]. HES increased the need for RRT and blood products during cardiac surgery [30]. Triggered by safety concerns from large-scale RCTs, the European Medicines Agency (EMA) re-assessed the safety and efficacy of HES solutions and decided to restrict its use in critically ill patients. In other settings, HES should only be considered for the treatment of hypovolaemia caused by acute blood loss if crystalloids are considered to be insufficient. In addition, HES is contraindicated in patients with impaired renal function and in patients with severe coagulopathy. The EMA also requested post-marketing studies to be carried out in surgical and trauma patients [31]. Several European anesthesiological societies have published comments regarding these EMA statements [32], [33].

Limitation of our survey include (1) lack of a prospective study design due a focused survey, (2) limited spectrum of questions within a postal survey and (3) the fact that the survey is mainly based on answers by one or few members of the cardiac anesthesia team rather than the whole team involved in cardiac surgery care of the respective institutions.

### Conclusion

This questionnaire focused on basic and regional monitoring, catecholamine as well as volume therapy among patients undergoing cardiac surgery in Germany. This survey shows a highly standardized basic hemodynamic monitoring among all participating centers. Regional perfusion monitoring, especially cerebral oximetry, is used as an additional monitoring. Catecholamine therapy in the treatment of LCOS is heterogeneous and therefore further clinical research as well as the development of clinical guidelines is warranted. Momentarily synthetic colloid fluids are a common part of cardiac anesthesia procedures in Germany. In the light of potential risk factors associated with synthetic colloids further clinical research is also urgently needed.

## Supporting Information

File S1 Questionnaire hemodynamic monitoring, catecholamine and volume therapy in cardiac surgery patients.
(DOC)

File S2 Distinctions in hemodynamic monitoring, catecholamine- and volume therapy among different levels of hospital care - Tables and Figures - Table S1: Availability of devices for intraoperative macrohemodynamic control/global perfusion monitoring among different levels of hospital care. Table S2: frequency of the intraoperative use of hemodynamic monitoring devices among different levels of hospital care. Table S3: Availability of special monitoring devices for regional perfusion control or for oxygen consumption among different levels of hospital care. Table S4: Frequency of the intraoperative use of the regional perfusion monitoring devices among different levels of hospital care. Table S5: Influence of catecholamine therapy by others (cardiac surgery, pharmacy, controlling). Table S6: Intraoperative application of colloidal fluids among different levels of hospital care. Table S7: Priming solution for cardiopulmonary bypass among different levels of hospital care. Table S8: Presence of a standard operating procedure for perioperative transfusion of packed red blood cells among different levels of hospital care. Figure S1: Percentage of catecholamine use in hospitals of different levels of care.
(DOC)
