# Supplementary material for: Catecholamine and Volume Therapy for Cardiac Surgery in Germany – Results from a Postal Survey
Source: PLoS One. 2014 Aug 1;9(8):e103996. doi: 10.1371/journal.pone.0103996 (PMC4118968; doi:10.1371/journal.pone.0103996)
Supplement: File S1 — Questionnaire hemodynamic monitoring, catecholamine and volume therapy in cardiac surgery patients. (DOC) [file pone.0103996.s001.doc]

**Catecholamine and volume therapy for cardiac surgery in Germany**

**– results from a postal survey**

**- Supporting Information File –I -**

Authors: Christoph Sponholz^1*^, Christoph Schelenz^1*^, Konrad Reinhart^1&2^, Uwe Schirmer^3^, Sebastian N. Stehr^1&2^

Affiliations: ^1^Department of Anesthesiology and Critical Care Medicine, University Hospital Jena, Germany

^2^Integrated Research and Treatment Center, Center for Sepsis Control and Care (CSCC), Jena University Hospital, Jena, Germany

^3^Institute of Anaesthesiology, Heart and Diabetes Center NRW, Ruhr University of Bochum, Bad Oeynhausen, Germany

**Corresponding author:** Christoph Sponholz

Department of Anesthesiology and Critical Care Medicine

Friedrich-Schiller-University Hospital

Erlanger Allee 101

D-07747 Jena

Germany

Phone: +49-3641-9322225

Fax: +49-3641-9323102

Mail: christoph.sponholz@med.uni-jena.de

**Questionnaire hemodynamic monitoring, catecholamine and volume therapy in cardiac surgery patients**

**1. Structural hospital data**

Total bed capacity: _____

□ heart center

□ maximal care hospital□

□ university hospital

**2. Number of annual cardiac surgery procedures (not including short procedures like pacemaker- or ICD implantation, wound debridement etc.)**

□ less than 750 procedures per year

□ between 750 and 1000 procedures per year

□ between 100 and 1500 procedures per year

□ between 1500 and 2000 procedures per year

□ more than 2000 procedures per year

with CPB _____ %

sole coronary surgery _____ %

**3. Management of postoperative intensive care?**

□ Anaesthesiology

□ Cardiac surgery

□ Interdisciplinary

**4. Standard operating procedure (SOP) for perioperative catecholamine use ?**

intraoperative □ yes □ no

postoperative □ yes □ no □ unkown (in case of surgical postoperative intensive care)

**5. Lower mean arterial- or systolic blood pressure limit, prior to intervention (volume challenge or catecholamine use)**

□ yes mean arterial- or systolic blood pressure _____ mmHg

□ yes, but in relation to comorbidities (e.g. carotid stenosis, long-lasting arterial

hypertension)

Please provide the lowest tolerated mean arterial- or systolic blood pressure without presence of comorbidities ____ mmHg

Please provide the lowest tolerated mean arterial- or systolic blood pressure with presence of comorbidities ____ mmHg

□ no

**6. Which monitoring devices for intraoperative macrohemodynamic control/global perfusion are available (multiple answers possible)?**

□ transesophageal echocardiography

□ PA Catheter

□ calibrated trend monitoring device (e.g. PiCCO)

□ non-calibrated trend monitoring device (e.g. Vigileo)

□ esophageal doppler (e.g. CardioQ)

□ other: _______

□ none or seldom intraoperative monitoring

**7. Please provide the frequency of the** intraoperative use of the following hemodynamic monitoring devices

|  | Is always present |  |  |  | Is never present |
| --- | --- | --- | --- | --- | --- |
|  | 1 | 2 | 3 | 4 | 5 |
| Basic monitoring (e.g. invasive arterial blood pressure) |  |  |  |  |  |
| Central venous pressure |  |  |  |  |  |
| Transesophageal echocardiography |  |  |  |  |  |
| PA Catheter |  |  |  |  |  |
| calibrated trend monitoring device (e.g. PiCCO) |  |  |  |  |  |
| non-calibrated trend monitoring device (e.g. Vigileo) |  |  |  |  |  |
| esophageal doppler (e.g. CardioQ) |  |  |  |  |  |
| other: _______ |  |  |  |  |  |

**8. Is a special monitoring device for regional perfusion control or for oxygen consumption available?**

□ yes

□ cerebral oxymetry

□ gastric tonometry

□ continuous central venous ScvO2

□ continuous mixed venous SvO2

□ other: ______

□ no

**9. Please provide the frequency of the** intraoperative use of the regional perfusion monitoring devices

|  | Is always present |  |  |  | Is never present |
| --- | --- | --- | --- | --- | --- |
|  | 1 | 2 | 3 | 4 | 5 |
| cerebral oxymetry |  |  |  |  |  |
| gastric tonometry |  |  |  |  |  |
| continuous central venous ScvO2 |  |  |  |  |  |
| continuous mixed venous SvO2 |  |  |  |  |  |
| other: ______ |  |  |  |  |  |

10. Volume therapy is mainly performed

|  | Is always present |  |  |  | Is never present |
| --- | --- | --- | --- | --- | --- |
|  | 1 | 2 | 3 | 4 | 5 |
| Clinically (e.g. pulse pressure variation) |  |  |  |  |  |
| Based on filling pressures    a) CVP  b) wedge pressure |  |  |  |  |  |
| By transesophageal echocardiography |  |  |  |  |  |
| By calibrated or non-calibrated trend monitoring devices |  |  |  |  |  |
| By diuresis |  |  |  |  |  |

**11. According to the CAPS-Care Study (J Card Surg 2011): How often is a catecholamine therapy initiated in cardiac surgery patients ?**

□ in 80 - 100 % of all patients

□ in 60 - 80 % of all patients

□ in 40 - 60 % of all patients

□ in less than 35 % of all patients

**12. Which is the first line catecholamine in hypotensive patients caused by low cardiac output syndrome?**

□ Cafedrine/Theodrenaline

□ Dopamine

□ Dobutamine

□ Ephedrine

□ Epinephrine

□ Phenylephrine

□ Norepinephrine

□ Phosphodiesterase Inhibitors

□ Levosimendan

□ Vasopressin

□ Methylene blue

**13. Which is the first line catecholamine in hypotensive patients caused by vasoplegia (e.g. under ACE-inhibitor therapy or SIRS)?**

□ Cafedrine/Theodrenaline

□ Dopamine

□ Dobutamine

□ Ephedrine

□ Epinephrine

□ Phenylephrine

□ Norepinephrine

□ Phosphodiesterase inhibitors

□ Levosimendan

□ Vasopressin

□ Methylene blue

**14. In case of no or only marginal success and optimal volume balance or under first line catecholamine therapy, which second line catecholamine is used?**

□ None

□ Cafedrine/Theodrenaline

□ Dopamine

□ Dobutamine

□ Ephedrine

□ Epinephrine

□ Phenylephrine

□ Norepinephrine

□ Phosphodiesterase inhibitors

□ Levosimendan

□ Vasopressin

□ Methylene blue

□ Oher: ____________

**15. Is or was your catecholamine therapy influenced by others (cardiac surgery, pharmacy, controlling) ?**

□ no

□ yes

**16. Colloids are used intraoperatively ....**

□ (nearly) always □ often □ less often □ never

**17. Which is the standard intraoperative colloidal fluid?**

□ Albumine

□ HES products

□ Gelatine

□ Fresh frozen plasma (FFP)

□ none, we always use crystalloids

**18. How frequently do you use colloids intraoperatively?**

|  | always |  |  |  | never |
| --- | --- | --- | --- | --- | --- |
|  | 1 | 2 | 3 | 4 | 5 |
| Albumine |  |  |  |  |  |
| HES |  |  |  |  |  |
| Gelatine |  |  |  |  |  |
| FFP |  |  |  |  |  |
| Only crystalloids |  |  |  |  |  |

**19. Is the CPB also primed with colloids ?**

□ yes, with ...

□ Albumine

□ HES

□ Gelatine

□ no

**20. Presence of a standard operating procedure (SOP) for perioperative transfusion of packed red blood cells?**

intraoperative □ yes □ no

postoperative □ yes □ no □ unkown (in case of surgical postoperative intensive care)

**21. How often do you use colloids in the postoperative period?**

|  | always |  |  |  | never |
| --- | --- | --- | --- | --- | --- |
|  | 1 | 2 | 3 | 4 | 5 |
| Albumine |  |  |  |  |  |
| HES |  |  |  |  |  |
| Gelatine |  |  |  |  |  |
| FFP |  |  |  |  |  |
| Only crystalloids |  |  |  |  |  |

**22. Regular use of Multiplate in patients under double platelet inhibition?**

□ yes

□ no

**23. Do you regularly discontinue a double platelet inhibition prior to elective cardiac surgery ?**

□ yes, for _____ days

□ no
