# Supplementary material for: Catecholamine and Volume Therapy for Cardiac Surgery in Germany – Results from a Postal Survey
Source: PLoS One. 2014 Aug 1;9(8):e103996. doi: 10.1371/journal.pone.0103996 (PMC4118968; doi:10.1371/journal.pone.0103996)

**Catecholamine and volume therapy for cardiac surgery in Germany**

**– results from a postal survey**

**- Supporting Information File-II -**

Authors: Christoph Sponholz^1*^, Christoph Schelenz^1*^, Konrad Reinhart^1&2^, Uwe Schirmer^3^, Sebastian N. Stehr^1&2^

Affiliations: ^1^Department of Anesthesiology and Critical Care Medicine, University Hospital Jena, Germany

^2^Integrated Research and Treatment Center, Center for Sepsis Control and Care (CSCC), Jena University Hospital, Jena, Germany

^3^Institute of Anaesthesiology, Heart and Diabetes Center NRW, Ruhr University of Bochum, Bad Oeynhausen, Germany

Corresponding author: Christoph Sponholz

Department of Anesthesiology and Critical Care Medicine

Friedrich-Schiller-University Hospital

Erlanger Allee 101

D-07747 Jena

Germany

Phone: +49-3641-9322225

Fax: +49-3641-9323102

Mail: christoph.sponholz@med.uni-jena.de

Distinctions in hemodynamic monitoring, catecholamine- and volume therapy among different levels of hospital care

- Tables and Figures -

Table S1: Availability of devices for intraoperative macrohemodynamic control/global perfusion monitoring among different levels of hospital care

|  | Transesophageal echocardiography | PA Catheter | Calibrated trend monitoring device | Non-calibrated trend monitoring device | Esophageal doppler probe |
| --- | --- | --- | --- | --- | --- |
| Heart center, n(%) | 16 (100) | 15 (93.8) | 6 (37.5) | 4 (25) | 0 (0) |
| University Hospital, n(%) | 18 (100) | 17 (94.4) | 13 (72.2) | 6 (33.3) | 0 (0) |
| Maximal Care Hospital, n(%) | 15 (100%) | 14 (93.3) | 11 (73.3) | 3 (20) | 1 (6.7) |

Table S2: frequency of the intraoperative use of hemodynamic monitoring devices among different levels of hospital care

|  | | Basic monitoring | Central venous pressure | Transesophageal echocardiography | PA Catheter | Calibrated trend monitoring device | Non-calibrated trend monitoring device | Esophageal doppler probe |
| --- | --- | --- | --- | --- | --- | --- | --- | --- |
| Heart center | N | 16 | 16 | 16 | 16 | 14 | 14 | 13 |
|  | Median (IQR) | 1 (1 – 1) | 1 (1 - 1) | 2 (1 – 2.75) | 3.5 (3 – 4) | 5 (4 – 5) | 5 (5 -5) | 5 (5 – 5) |
| University Hospital | N | 18 | 18 | 18 | 18 | 15 | 17 | 14 |
|  | Median (IQR) | 1 (1 – 1) | 1 (1 – 1) | 2 (1 – 2) | 3 (3 – 4) | 4 (3 – 5) | 5 (4 – 5) | 5 (5 – 5) |
| Maximal Care Hospital | N | 15 | 15 | 15 | 15 | 14 | 13 | 11 |
|  | Median (IQR) | 1 (1 – 1) | 1 (1 – 1) | 2 (1 – 3) | 3 | 4 (3.75 – 5) | 5 (4.5 – 5) | 5 (5 – 5) |

Table S3: Availability of special monitoring devices for regional perfusion control or for oxygen consumption among different levels of hospital care

|  | cerebral oximetry | continuous central venous ScvO2 | continuous mixed venous SvO2 | No regional perfusion device |
| --- | --- | --- | --- | --- |
| Heart center, n(%) | 11 (68.8) | 1 (6.3) | 3 (18.8) | 4 (25.0) |
| University Hospital, n(%) | 15 (83.3) | 4 (22.2) | 9 (50.0) | 1 (5.6) |
| Maximal Care Hospital, n(%) | 10 (66.7) | 1 (6.7) | 4 (26.7) | 4 (26.7) |

Table S4: Frequency of the intraoperative use of the regional perfusion monitoring devices among different levels of hospital care

|  |  | cerebral oximetry | gastric tonometry | continuous central venous ScvO2 | continuous mixed venous SvO2 |
| --- | --- | --- | --- | --- | --- |
| Heart center | N | 14 | 8 | 9 | 10 |
|  | Median (IQR) | 3 (3 – 3.5) | 5 (5 – 5) | 5 (5 – 5) | 5 (3.75 – 5) |
| University Hospital | N | 17 | 14 | 14 | 16 |
|  | Median (IQR) | 3 (1.5 – 4) | 5 (5 – 5) | 5 (4 – 5) | 4 (3.25 – 5) |
| Maximal Care Hospital | N | 14 | 10 | 11 | 12 |
|  | Median (IQR) | 3 (3 – 5) | 5 (5 – 5) | 5 (4 – 5) | 5 (4 – 5) |

Table S5: Influence of catecholamine therapy by others (cardiac surgery, pharmacy, controlling)

|  | Heart center, n(%) | University Hospital, n(%) | Maximal Care Hospital, n(%) |
| --- | --- | --- | --- |
| Yes | 1 (6.3) | 4 (22.2) | 1 (6.7) |
| No | 15 (93.8) | 14 (77.8) | 14 (93.3) |

Table S6: Intraoperative application of colloidal fluids among different levels of hospital care

|  | Heart center, n(%) | University Hospital, n(%) | Maximal Care Hospital, n(%) |
| --- | --- | --- | --- |
| (nearly) always |  | 4 (22.2) | 1 (6.7) |
| often | 7 (43.8) | 4 (22.2) | 4 (26.7) |
| less often | 8 (50.0) | 6 (33.3) | 9 (60.0) |
| never | 1 (6.3) | 4 (22.2) | 1 (6.7) |

Table S7: Priming solution for cardiopulmonary bypass among different levels of hospital care

|  | Heart center, n(%) | University Hospital, n(%) | Maximal Care Hospital, n(%) |
| --- | --- | --- | --- |
| Albumine | 1 (6.3) |  | 1 (6.7) |
| HES | 6 (37.5) | 7 (38.9) | 3 (20.0) |
| Gelatine |  | 4 (22.2) | 1 (6.7) |
| No colloidal fluids | 9 (56.3) | 7 (38.9) | 10 (66.7) |

Table S8: Presence of a standard operating procedure for perioperative transfusion of packed red blood cells among different levels of hospital care

|  | | Heart center, n(%) | | University Hospital, n(%) | | Maximal Care Hospital, n(%) | |
| --- | --- | --- | --- | --- | --- | --- | --- |
| Intraoperative | Yes | 13 (81.3) | | 9 (50.0) | | 6 (40.0) | |
|  | No | 3 (18.8) | | 9 (50.0) | | 9 (60.0) | |
|  |  |  |  |  |  |  |  |
| Postoperative | Yes | 12 (75.0) | | 7 (38.9) | | 2 (13.3) | |
|  | No | 3 (18.8) | | 7 (38.9) | | 11 (73.3) | |
|  | Unknown | 1 (6.3) | | 3 (16.7) | | 1 (6.7) | |

Figure S1: Percentage of catecholamine use in hospitals of different levels of care


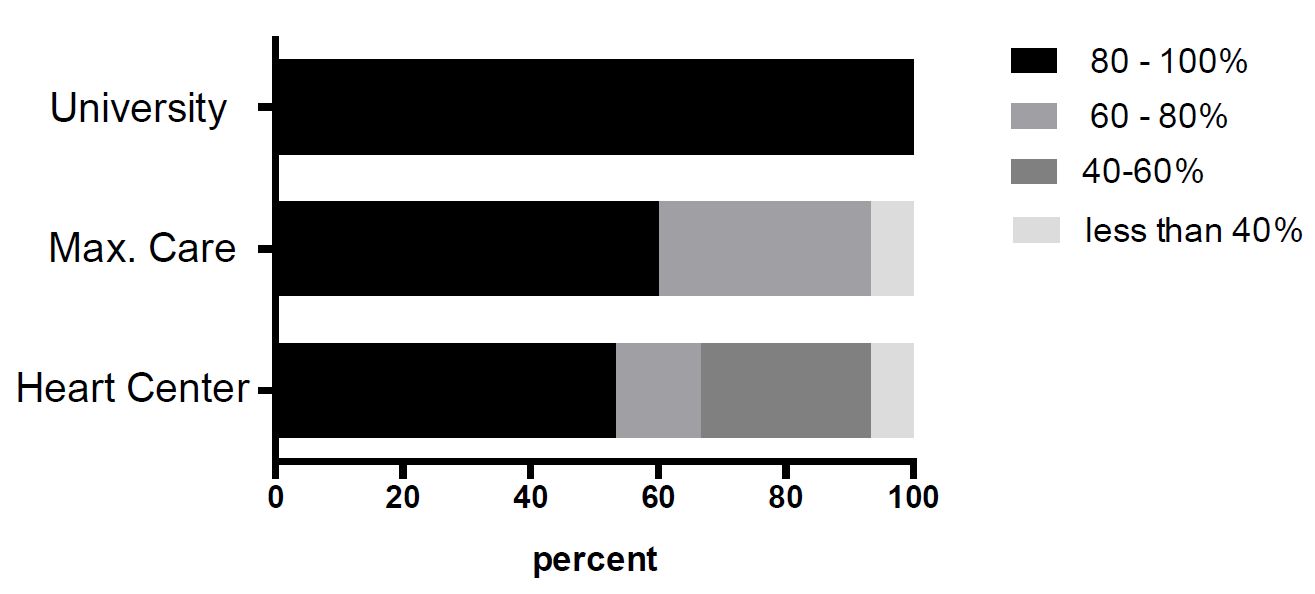

Supplement: File S2 — Distinctions in hemodynamic monitoring, catecholamine- and volume therapy among different levels of hospital care - Tables and Figures - Table S1: Availability of devices for intraoperative macrohemodynamic control/global perfusion monitoring among different levels of hospital care. Table S2: frequency of the intraoperative use of hemodynamic monitoring devices among different levels of hospital care. Table S3: Availability of special monitoring devices for regional perfusion control or for oxygen consumption among different levels of hospital care. Table S4: Frequency of the intraoperative use of the regional perfusion monitoring devices among different levels of hospital care. Table S5: Influence of catecholamine therapy by others (cardiac surgery, pharmacy, controlling). Table S6: Intraoperative application of colloidal fluids among different levels of hospital care. Table S7: Priming solution for cardiopulmonary bypass among different levels of hospital care. Table S8: Presence of a standard operating procedure for perioperative transfusion of packed red blood cells among different levels of hospital care. Figure S1: Percentage of catecholamine use in hospitals of different levels of care. (DOC) [file pone.0103996.s002.doc]
